# Potential Health Disparities in the Early Detection and Prevention of Pancreatic Cancer

**DOI:** 10.7759/cureus.60240

**Published:** 2024-05-13

**Authors:** Michele T Yip-Schneider, Rodica Muraru, Nikita Rao, Rachel C Kim, Jennifer Rempala-Kurucz, Jackson A Baril, Alexandra M Roch, C. Max Schmidt

**Affiliations:** 1 Surgery, Indiana University School of Medicine, Indianapolis, USA; 2 Pancreatic Cyst and Cancer Early Detection Center, Indiana University Health, Indianapolis, USA; 3 Center for Outcomes Research in Surgery, Indiana University School of Medicine, Indianapolis, USA; 4 Surgery, Biochemistry/Molecular Biology, Indiana University School of Medicine, Indianapolis, USA; 5 Simon Comprehensive Cancer Center, Indiana University, Indianapolis, USA

**Keywords:** early detection, survey, biobanking, prevention, pancreatic cysts, pancreatic cancer, racial disparity

## Abstract

Introduction: Pancreatic cancer remains one of the deadliest cancers in the United States. Some types of pancreatic cysts, which are being detected more frequently and often incidentally on imaging, have the potential to develop into pancreatic cancer and thus provide a valuable window of opportunity for cancer interception. Although racial disparity in pancreatic cancer has been described, little is known regarding health disparities in pancreatic cancer prevention. In the present study, we investigate potential health disparities along the continuum of care for pancreatic cancer.

Methods: The racial and ethnic composition of pancreatic patients at high-volume centers in Indiana were evaluated, representing patients undergoing surgery for pancreatic cancer (n=390), participating in biobanking (972 pancreatic cancer patients and 1984 patients with pancreatic disease), or being monitored for pancreatic cysts at an early detection center (n=1514). To assess racial disparities and potential differences in decision-making related to pancreatic cancer prevention and early detection, an exploratory online survey was administered through a volunteer registry (n=708).

Results: We show that despite comprising close to 10% or 30% of the Indiana or Indianapolis population, respectively, African Americans make up only about 4-5% of our study cohorts consisting of patients undergoing pancreatic surgery or participating in biobanking and early detection. Analysis of online survey results revealed that given the hypothetical situation of being diagnosed with a pancreatic cyst or pancreatic cancer, the vast majority of respondents (>90%) would agree to undergo surveillance or surgery, respectively, regardless of race. Only a minority (3-12%) acknowledged any significant transportation, financial, or emotional barriers that would impact a decision to undergo surveillance or surgery. This suggests that the observed racial disparities may be due in part to the existence of other barriers that lie upstream of this decision point.

Conclusion: Racial disparities exist not only for pancreatic cancer but also at earlier points along the continuum of care such as prevention and early detection. To our knowledge, this is the first study to document racial disparity in the management of patients with pancreatic cysts who are at risk of developing pancreatic cancer. Our results suggest that improving access to information and care for such at-risk individuals may lead to more equitable outcomes.

## Introduction

Pancreatic cancer remains one of the deadliest cancers in the United States with a five-year relative survival rate that has slowly improved to 12%. According to the American Cancer Society, there will be an estimated 66,440 new diagnoses and 51,750 deaths from pancreatic cancer in 2024 [1]. The most recent annual age-adjusted incidence and death rates for pancreatic cancer are 13.4 and 11.1, respectively, per 100,000 people in the United States [1]. Further stratification by race reveals incidence rates of 16.2 for non-Hispanic Black, 13.8 for non-Hispanic White, 12.1 for Hispanic, and 10.1 for Asian/Pacific Islander populations per 100,000 people [2]. The death rate is also higher for non-Hispanic Blacks than any other racial group in the United States at 13.6 per 100,000, suggesting potential racial disparities along the continuum of pancreatic cancer care that could lead to worse outcomes.

To date, surgery is the only curative treatment option for patients diagnosed with resectable cancer (approximately one in five patients). Earlier points along the continuum of care include prevention and early detection of pancreatic cancer. Certain types of pancreatic cysts have been identified as precursors of pancreatic cancer. Importantly, pancreatic cysts are being detected more frequently on radiological imaging, often incidentally due to an unrelated medical reason, thus offering a window of opportunity for cancer interception. Clinical management of such patients is challenging and involves distinguishing pancreatic cancer or pancreatic cysts at high risk of malignant progression, which should be resected in fit patients, from benign or low/moderate-risk pancreatic cysts, which may be safely monitored by annual or semi-annual abdominal imaging and bloodwork for risk stratification [3].

In the state of Indiana, the pancreatic cancer incidence and death rates are 13.4 and 12 per 100,000 people, respectively, higher than the national averages in 2023 [4]. As observed at the national level, the death rate for African Americans is higher than other racial groups in Indiana at 14 per 100,000, consistent with racial disparity in pancreatic cancer outcomes [5]. Although racial disparity in pancreatic cancer and possible contributing factors have been described in the literature [6-9], little is known about health disparities in pancreatic cancer prevention. High-volume centers for the management of patients with pancreatic disease, including pancreatic cancer and pancreatic cysts, exist within the statewide Indiana University (IU) Health system and provide patient populations that can inform clinical and translational research.

In the present study, we sought to further examine health disparities related to pancreatic cancer by evaluating the racial distribution of pancreatic patients within the IU Health network. These cohorts represent patients undergoing surgery for pancreatic cancer, biobanking of samples from patients with pancreatic disease, and patients at increased risk of pancreatic disease being monitored at an early detection clinic. An exploratory online survey was also administered to volunteers to assess differences in personal healthcare decision-making that might affect downstream choices regarding the prevention and early detection of pancreatic cancer. Questions addressing potential barriers to care were included to provide preliminary insight into factors that may impact health outcomes for diverse, historically underrepresented communities. Strategies targeting such factors would be important early steps towards addressing pancreatic cancer inequities.

## Materials and methods

Pancreatic cancer and pancreatic cyst patients

Patient race/ethnicity data (White, Black/African American, Asian, Hispanic, Other (American Indian, Hawaiian/Pacific Islander, multiracial, unknown)) was obtained from the following: (i) retrospective review of a prospectively maintained institutional database identifying all patients who underwent surgery for pancreatic adenocarcinoma, confirmed on surgical pathology, at IU Health University Hospital (January 2016-February 2020), (ii) information requested about patient pancreatic cancer samples collected from 2004 to September 2023 through the Biospecimen Collection and Banking Core at the IU Simon Comprehensive Cancer Center, (iii) database consisting of patients at increased risk of pancreatic cancer (new or existing) identified for monitoring at the IU Health Pancreatic Cyst and Cancer Early Detection Center (PCC-EDC) from 2020 to April 2023, and (iv) database of outpatient and surgical patients from whom samples were collected through the IU Pancreatic Tissue Fluid Bank (2003 to September 2023).

Survey design

An exploratory online survey was designed to capture information about factors that might influence health decision-making and consisted of questions about (i) personal healthcare, (ii) pancreatic cancer experience/awareness, (iii) two hypothetical situations and potential barriers to care, and (iv) demographics (age, sex/gender, race/ethnicity, residence zip code, total annual household income, highest level of education completed). Responses were multiple choice (two to five choices); the option “prefer not to answer” was offered for the demographic questions. At the end of the survey, respondents were given the opportunity to provide additional explanations in an open text space. The study was approved by the Indiana University Institutional Review Board (approval number: 18713).

Survey distribution and data collection

The online survey was built using REDCap (Research Electronic Data Capture), a secure, web-based software platform designed to support data capture for research studies and hosted at IU [10,11]. Email invitations to complete the survey were distributed to the All IN for Health Volunteer Registry through the Indiana Clinical and Translational Sciences Institute (CTSI) at the IU School of Medicine (IUSM) in July 2023 (13,410 emails sent). Participants were required to be >18 years old and residents of the United States. No incentive was offered. A second email reminder was sent after four weeks to registry volunteers identifying as underrepresented groups (non-White). The survey was open for eight weeks during which time participant responses were collected anonymously using REDCap. Respondents could skip any question they did not want to answer. This study was considered exempt and conducted in compliance with the IU Institutional Review Board.

Data analysis

Surveys missing racial data were excluded from the analysis. Missing data in the surveys was less than 5% for the selected variables so the dataset was used without any modification. For race, respondents self-identifying as White/Caucasian alone were classified as “White/Caucasian”; those self-identifying as “Black/African American” were counted in this group even if they selected options in addition to Black/African American. All others self-identifying as another racial/ethnic group were placed in the specific group. For insurance, respondents were classified as follows: none (no insurance), private (private only, private/other), Medicare (Medicare only, Medicare/private, Medicare/other), Medicaid (Medicaid only, Medicaid/Medicare, Medicaid/other). For factors influencing healthcare decisions, answers were collated into none only, friends and/or family, culture and/or religion, or combined (more than two choices selected). For multiple-choice responses or those using the Likert scale, variables were collapsed to facilitate analysis as indicated. Respondents’ residence zip codes were classified as rural, mixed, or urban. In-state zip codes were assigned to a county and classified using a method developed by Purdue University based on population size/density, the size of the largest city/town in the county, and the “identity” that people had of their county [12,13]. Out-of-state zip codes were classified using the United States Department of Agriculture’s rural-urban commuting area (RUCA) codes based on population density, urbanization, and daily commuting [14]. Percentages were calculated using the total number of participants responding to each question as the denominator to include the maximum number of submitted surveys/responses.

To compare Black/African American and White study cohorts, bivariate analysis (Fisher’s exact test if the sample size was less than five or Chi-square test) was performed followed by ordinal regression analysis (gologit2) for outcomes of particular interest with p-value <0.05 (Stata Statistical Software: Release 16 (2019); StataCorp LLC, College Station, Texas, United States). Statistical significance was defined as a p-value < 0.05.

## Results

To assess potential health disparities related to pancreatic cancer, we evaluated the racial distribution of four groups of patients served in Indiana (Table 1). IU Health is a statewide integrated healthcare system comprised of 17 hospitals. Race data for the first cohort was obtained from patients who underwent surgery for confirmed pancreatic adenocarcinoma at IUH University Hospital, the largest hospital in Indiana, located in Indianapolis within Marion County, which is the hospital’s primary service area. The second set of race/ethnicity data was obtained from pancreatic cancer patients with biospecimens banked through the IU Melvin and Bren Simon Comprehensive Cancer Center, a National Cancer Institute (NCI)-designated comprehensive cancer center within the IU Health network. The third set of data was gathered from patients at increased risk of developing pancreatic cancer being monitored at the PCC-EDC, part of IU Health and the IU School of Medicine. Finally, the fourth group consisted of patients who underwent surgery or were under surveillance for pancreatic disease and for whom biospecimens were banked through the Pancreatic Tissue Fluid Bank associated with the PCC-EDC. In all four cohorts, White patients represented the majority at >90% of the total. In contrast, Black/African American patients comprised <5% of the total, while Asians or Hispanics were <1.5%, substantially lower than the percentages of each in Indianapolis or Indiana as estimated by the United States Census Bureau for the inclusive years (Table 1) [15-18].

**Table 1:** Racial and ethnic composition of Indiana study cohorts compared to United States census data *Other - American Indian/Alaska Native, Native Hawaiian/Pacific Islander, multiracial, or unknown Percent (%) rounded to nearest integer if >10% IU, Indiana University

Cohort	Time Period	Racial/Ethnic Composition, n (% total)					Total patients
		White	Black/African American	Asian	Hispanic/Latino	Other*	
IU Health University Hospital							
Patients undergoing surgery for pancreatic cancer	2016-2020	359 (92%)	18 (4.6%)	5 (1.3%)	-	8 (2.1%)	390
IU Melvin and Bren Simon Cancer Center Bank							
Pancreatic cancer patients with banked samples	2004-2023	903 (93%)	39 (4.0%)	3 (0.3%)	9 (0.9%)	18 (1.8%)	972
IU Pancreatic Cyst and Cancer Early Detection Center							
New or existing patients at risk of pancreatic cancer	2020-2023	1361 (90%)	63 (4.1%)	23 (1.5%)	22 (1.5%)	45 (2.9%)	1514
IU Pancreatic Tissue Fluid Bank							
Patients with pancreatic disease and banked samples	2003-2023	1834 (92%)	99 (5.0%)	22 (1.1%)	10 (0.5%)	19 (0.9%)	1984
United States Census Bureau							Population
Indianapolis population	2000	65%	26%	1.4%	3.9%	3.9%	781,870
	2022	53%	29%	3.9%	10%	5.6%	880,621
Indiana population	2003	84%	8.8%	1.2%	4.5%	1.3%	6,237,569
	2022	77%	10%	2.8%	7.9%	2.9%	6,833,037

Taken together and considering the higher pancreatic cancer incidence and death rates for the Black/African American population compared with the White population in the United States, the percentage of Black/African American patients in all four Indiana cohorts was surprisingly low. Our results thus suggest racial disparities in the numbers of patients who undergo resection for pancreatic cancer, are represented in biobanks, or are being monitored for precancerous pancreatic cysts. This led us to conduct an exploratory survey to investigate whether differences in healthcare decision-making may play a role in racial/ethnic disparities related to the pancreatic cancer care continuum.

A total of 712 participants responded to an online survey administered through an Indiana-based volunteer registry over an eight-week period, corresponding to a response rate of 5.3%. Demographic information including race/ethnicity, gender, age, highest level of education completed, total annual household income, and residence zip code were gathered (Table 2). The majority of respondents were White/Caucasian (79%), female (77%), 50-69 years old (47%), college educated (81%), and lived in an urban area based upon zip code (85%). Total annual household income was fairly evenly distributed, ranging from less than $35,000 to above $150,000. Further analysis was then performed to assess differences between the Black/African American and White subgroups of interest for the present study (Table 2). Significant associations between race and education, income, and residence classification were observed in the bivariate analysis (p=0.004, <0.001, and 0.007, respectively). Specifically, a higher percentage of Black/African American compared to White respondents had a lower level of education (<high/technical school: 19/14% vs 9/7%) as well as income (<$35,000: 29% vs 11%) and lived in an urban area (95% vs 83%) (Table 2).

**Table 2:** Survey demographics of entire cohort and race subgroup analysis ^a^Other - self-identify as American Indian, Hawaiian/Pacific Islander, other Percent (%) rounded to nearest integer if >10%; total indicates number of participants responding to each question *Chi-square test or **Fisher's exact test performed

Demographics	Entire Survey Cohort, n (%)	Black/African American, n (%)	White/Caucasian, n (%)	Black/African American vs White, p-value
Race/Ethnicity				
White/Caucasian	566 (79%)		566 (100%)	
Black/African American	91 (13%)	91 (100%)		
Asian	29 (4.1%)			
Hispanic/Latino	20 (2.8%)			
Other^a^	6 (0.8%)			
Total	712 (100%)			
Gender				0.74*
Female	535 (77%)	67 (75%)	427 (77%)	
Male	164 (23%)	22 (25%)	129 (23%)	
Total	699 (100%)	89 (100%)	556 (100%)	
Age (years)				0.06*
18-29	72 (10%)	6 (6.6%)	51 (9.0%)	
30-49	176 (25%)	33 (36%)	131 (23%)	
50-69	336 (47%)	38 (42%)	271 (48%)	
>70	128 (18%)	14 (15%)	113 (20%)	
Total	712 (100%)	91 (100%)	566 (100%)	
Highest education completed				0.004*
High school or less	73 (10%)	17 (19%)	52 (9.3%)	
Technical school	58 (8.3%)	12 (14%)	41 (7.3%)	
Undergraduate	249 (35%)	26 (30%)	209 (37%)	
Graduate/professional	322 (46%)	33 (38%)	257 (46%)	
Total	702 (100%)	88 (100%)	559 (100%)	
Annual Household Income				<0.001**
Less than $35,000	93 (14%)	23 (29%)	59 (11%)	
35-59,000	113 (17%)	16 (20%)	88 (17%)	
60-99,000	170 (26%)	19 (24%)	142 (27%)	
100-150,000	144 (22%)	16 (20%)	118 (23%)	
>150,000	128 (20%)	5 (6.3%)	110 (21%)	
Total	648 (100%)	79 (100%)	517 (100%)	
Residence classification				0.007**
Urban	582 (85%)	80 (95%)	452 (83%)	
Mixed	71 (10%)	2 (2.4%)	66 (12%)	
Rural	29 (4.2%)	2 (2.4%)	27 (5.0%)	
Total	682 (100%)	84 (100%)	545 (100%)	

Survey questions assessed respondents’ personal healthcare (medical check-ups/insurance status), experience with/awareness of pancreatic cancer, and decision-making if they were to be diagnosed with either a pancreatic cyst or pancreatic cancer (Table 3). The majority of respondents had a medical doctor they saw regularly (94%), annual medical check-ups (82%), and some type of health insurance (99%). Only 1% of respondents had pancreatic cancer themselves, 55% knew family or friends who had pancreatic cancer, but 44% did not personally know anyone with pancreatic cancer. Only 8% stated that they were very aware of risk factors for pancreatic cancer, with the remainder either slightly/moderately aware (47%) or not aware at all (45%), thus revealing a substantial knowledge gap. The majority did not think that the number of people diagnosed with pancreatic cancer differed by race (57%); 32% thought race was a factor. Especially relevant for the present study, given the hypothetical situation of being diagnosed with a pancreatic cyst and asked whether they would agree to have annual imaging and bloodwork done for monitoring at an early detection clinic, the vast majority (95%) replied that they would be likely or extremely likely to agree. Asked if barriers such as difficulty getting to appointments (i.e., work hours, needing transportation), financial concerns (i.e., cost, lack of insurance coverage), or emotions (i.e., fear, anxiety) would prevent them from being monitored annually at an early detection clinic, only a minority (3%, 12%, or 2%, respectively) responded that they would be affected by these to a large/great extent. Very similar results were obtained for a hypothetical diagnosis of pancreatic cancer and agreement to recommended surgery. Finally, 45% said that family/friends influence their healthcare decisions, while 46% said that family, friends, religion, and culture are not factors in their decision-making process.

**Table 3:** Survey responses of entire cohort and race subgroup analysis Percent (%) rounded to nearest integer; Likert scale or multiple choice responses collapsed as indicated for analysis *Chi-square test, **Fisher's exact test or ***ordinal regression performed n, total number of respondents for each question in cohorts; Bl/AA, Black/African American

Responses	Entire survey cohort, % total	Bl/AA, % total	White, % total	Bl/AA vs White, association p-value	Collapsed variables	Bl/AA vs White, association p-value (collapsed variables)
Medical doctor seen regularly	n = 707 (100%)	n = 90 (100%)	n= 562 (100%)	0.63**		
No	6%	4%	6%			
Yes	94%	96%	94%			
Medical check-up frequency	n = 710 (100%)	n= 90 (100%)	n= 565 (100%)	0.034**		
Every year	82%	81%	83%		every year	0.67*
Once in 2-5 years	8%	3%	9%		other frequencies	
Once in 6-10 years/never	1%	0%	1%			
Only when needed	9%	16%	8%			
Health insurance	n = 711 (100%)	n = 91 (100%)	n= 565 (100%)	<0.001**		
Private/employer-sponsored	58%	38%	59%			
Medicaid	9%	34%	5%			
Medicare	30%	24%	33%			
Other	1%	0%	1%			
None	1%	3%	1%			
Personal experience with pancreatic cancer	n = 710 (100%)	n = 91 (100%)	n = 564 (100%)	<0.001**		
No	44%	52%	41%		no	0.06*
Self	1%	7%	1%		yes	
Family, friends	55%	42%	58%			
Awareness of pancreatic cancer risk factors	n = 712 (100%)	n = 91 (100%)	n = 566 (100%)	0.09*		
Not at all	45%	45%	45%			
Slightly/moderately aware	47%	42%	48%			
Very/extremely aware	8%	13%	7%			
Pancreatic cancer numbers and dependence on race?	n = 706 (100%)	n = 89 (100%)	n = 563 (100%)	<0.001*		
Don’t know	57%	36%	61%			
No	10%	20%	9%			
Yes	32%	44%	30%			
If diagnosed with cyst, annual surveillance?	n = 709 (100%)	n = 91 (100%)	n = 563 (100%)	0.13**		
Extremely unlikely/unlikely	3%	3%	2%			
Neutral	2%	4%	2%			
Likely/extremely likely	95%	92%	96%			
Difficulty getting to appointment (i.e., work, transport)	n = 711 (100%)	n = 91 (100%)	n = 566 (100%)	0.035*		0.55***
Not at all	73%	71%	75%			
Little/somewhat	25%	22%	24%			
Large/great extent	3%	7%	2%			
Financial concerns (i.e., cost, insurance coverage)	n = 706 (100%)	n = 89 (100%)	n = 564 (100%)	0.3*		
Not at all	54%	57%	56%			
Little/somewhat	34%	27%	33%			
Large/great extent	12%	16%	12%			
Emotional barriers (i.e., fear, anxiety)	n = 711 (100%)	n = 91 (100%)	n = 565 (100%)	<0.001*		
Not at all	74%	69%	76%			
Little/somewhat	24%	23%	23%			
Large/great extent	2%	8%	1%			
If diagnosed with pancreatic cancer, surgery?	n = 708 (100%)	n = 89 (100%)	n = 565 (100%)	1**		
Extremely unlikely/unlikely	5%	4%	5%			
Neutral	3%	2%	3%			
Likely/extremely likely	92%	93%	92%			
Financial concerns (i.e., cost, insurance coverage)	n = 711 (100%)	n = 91 (100%)	n = 565 (100%)	0.058*		
Not at all	59%	57%	61%			
Little/somewhat	32%	27%	31%			
Large/great extent	9%	15%	7%			
Emotional barriers (i.e., fear, anxiety)	n = 708 (100%)	n = 90 (100%)	n = 563 (100%)	<0.001*		
Not at all	68%	57%	70%			
Little/somewhat	30%	36%	29%			
Large/great extent	2%	8%	1%			
External factors influencing healthcare decisions	n = 710 (100%)	n = 90 (100%)	n = 565 (100%)	0.008*		
Family, friends	45%	33%	45%			
Religion, culture	2%	6%	1%			
Combination (family, friends, religion, culture)	8%	11%	7%			
None	46%	50%	46%			

Additional subgroup analysis was performed to explore differences between the Black/African American and White cohorts (Table 3). Relevant to the current study, when confronted with a hypothetical diagnosis of pancreatic cancer or potentially precancerous pancreatic cyst, no significant difference between the two cohorts was observed (p=1 or 0.13, respectively), suggesting that the lower-than-expected Black/African American representation in our four Indiana study cohorts does not appear to be due to differences in a conscious decision or specific choice made at that point in time. Since the overwhelming majority of either group would opt for surgery or surveillance, other upstream barriers may be responsible for the observed racial disparities along the continuum of care. Interestingly, of the minority who would not undergo surveillance or surgery, only a few acknowledged the financial/emotional barriers queried in the survey as being a deciding factor (data not shown).

We further examined two of the outcomes of particular interest shown to be significant in the bivariate analysis. A significant association was shown between race and medical check-up frequency (p=0.034) (Table 3). However, when the variables were collapsed to compare yearly check-ups versus other frequencies (once in two to five years or six to 10 years or as needed), there was no significant difference between the Black/African American and White cohorts (p=0.67). This suggests that both groups held similar attitudes toward the importance of annual check-ups and preventive healthcare. The second outcome was the potential barrier of difficulty getting to appointments. After performing a multivariable ordinal regression controlling for insurance status, education, and income, the association with race was no longer significant (p=0.55). Other significant outcomes of the bivariate analysis will require more in-depth research beyond the scope of this initial study to understand their impact on decision-making.

## Discussion

Pancreatic cancer is often discovered at an advanced, inoperable stage due to the lack of specific symptoms as well as diagnostic screening tests for the general population. Recent efforts have focused on management strategies that emphasize prevention and earlier detection to improve patient outcomes. Health disparities have emerged in several types of cancers including pancreatic, but little is known with respect to early detection [19]. To explore this in the present study, we investigated potential disparities along the pancreatic cancer care continuum by examining racial/ethnic data from high-volume pancreatic centers in Indiana. We show that despite comprising close to 10% or 30% of the Indiana or Indianapolis population, respectively, Black/African Americans were found to make up only about 4-5% of our study cohorts representing patients undergoing pancreatic surgery or participating in biobanking and early detection. This racial disparity apparent at multiple levels of care is similar to the low Black/African American relative to White participation reported previously for pancreatic cancer biobanking and clinical trials [20-22]. Reasons for lack of participation in clinical research studies among African Americans include historical medical distrust of the research community, fear of research, and lack of information [23]. Racial disparity has also been revealed for high-risk individuals (genetic susceptibility or family history of pancreatic cancer) undergoing surveillance for pancreatic cancer through the Pancreatic Cancer Early Detection (PRECEDE) consortium; specifically, 87.7% of high-risk individuals self-identified as White, and only 2% as Black (total 1113 subjects) [24]. To our knowledge, this is the first study to document racial disparity in the management of patients with pancreatic cysts.

To gain insight into whether differences in healthcare decision-making might be responsible for the observed racial disparities along the pancreatic cancer care continuum, an online survey of volunteers was performed. Interestingly, despite significant differences in demographics and survey responses between Black/African American and White subgroups, an overwhelming majority of respondents would undergo surveillance or surgery for pancreatic cysts or cancer if given the option, regardless of race. This is consistent with our finding of no significant difference between Black/African American and White respondents in having a medical doctor or annual medical check-ups, reflecting similar attitudes towards at least routine preventive healthcare. Furthermore, Black/African Americans have been shown to undergo preventive screenings for other cancer types (cervical, prostate, colorectal, breast) at the same or higher rates than White individuals in Indiana [25,26]. Overall, our findings suggest that the racial disparity observed in our Indiana pancreatic cohorts may be due to barriers further upstream; for example, lack of information about pancreatic cancer prevention and early detection, not being diagnosed or referred early enough or at all, due to inequities in access to care. In a survey of 100 pancreatic cyst patients, Verma et al. reported that 96% did not know their cyst type and 58% did not know the risk for malignant transformation associated with their cyst [27]. Racial disparities in the type and level of care received may further widen this knowledge gap for diverse groups. In a large study of the Surveillance, Epidemiology, and End Results Registry (1991-2002), Murphy et al. reported that African American patients with pancreatic cancer were less likely to consult a cancer specialist such as a medical oncologist, radiation oncologist, or surgeon, resulting in worse outcomes [28]. Additionally, pancreatic cancer patients undergoing resection at high-volume facilities have been shown to have better outcomes and lower mortality [29]; however, African American patients with pancreatic cancer are less likely to be treated at high-volume centers [7]. Although these reports are specific to pancreatic cancer, similar disparities in the type of care received may exist for pancreatic cyst patients as suggested by our findings.

To reduce these disparities and bridge the overall knowledge gap, community outreach and education are needed to increase awareness of the importance of early detection and participation in biobanking and clinical trials. More diverse representation in these avenues would improve outcomes as well as provide important resources for inclusive research. Additionally, patients may not be aware that most health insurance should cover the cost of pancreatic cyst surveillance (imaging/bloodwork), so this should be clearly communicated to alleviate financial concerns about long-term management. Finally, improved patient navigation through the continuum of care from primary care provider to a specialist with expertise in pancreatic cyst management would ensure timely diagnosis and accurate risk stratification of potentially precancerous cysts. Interestingly, in a study performed at a Kaiser Permanente integrated health system, no racial disparities in diagnosis, treatment, or outcomes of pancreatic cancer were observed, suggesting that equitable access to high-quality coordinated care can reduce disparities [30].

Limitations of this study include that conclusions are based on selected patient databases and the demographic responding to the survey, which may not be representative of the general population. Since the survey was online and anonymized, responses could not be further investigated by follow-up questions. The survey was exploratory in nature, allowing us to investigate potential relationships between decision-making and the continuum of pancreatic cancer care. However, further in-depth follow-up studies will be required to rigorously evaluate these relationships and confirm our conclusions.

## Conclusions

Racial disparities exist not only for pancreatic cancer but also at earlier points along the continuum of care such as prevention and early detection, as revealed in the present study. Lower than expected percentages of Black/African American patients compared to White patients, based upon racial composition of the catchment area, were found for those undergoing surgery for pancreatic cancer, participating in pancreas-related biobanking, and being monitored at an early detection center for potential precancerous pancreatic cysts. An online volunteer survey showed no difference in the decision to undergo surveillance or surgery if diagnosed with a pancreatic cyst or cancer, respectively, between Black/African American and White respondents. This suggests that factors upstream may influence patient decision-making and thus, improving access to information and care for at-risk individuals may lead to more equitable outcomes. 
